# Blood-based mitochondrial respiratory chain function in major depression

**DOI:** 10.1038/s41398-021-01723-x

**Published:** 2021-11-17

**Authors:** Johan Fernström, Synthia H. Mellon, Marlon A. McGill, Martin Picard, Victor I. Reus, Christina M. Hough, Jue Lin, Elissa S. Epel, Owen M. Wolkowitz, Daniel Lindqvist

**Affiliations:** 1 Lund University, Faculty of Medicine, Department of Clinical Sciences Lund, Psychiatry, Lund, Sweden; 2 Department of OB/GYN and Reproductive Sciences, University of California San Francisco (UCSF) School of Medicine, San Francisco, CA USA; 3 Division of Behavioral Medicine, Department of Psychiatry and Department of Neurology, H. Houston Merritt Center, Columbia Translational Neuroscience Initiative, Columbia University Irving Medical Center, New York, NY USA; 4 New York State Psychiatric Institute, New York, NY USA; 5 Weill Institute for Neurosciences/ Department of Psychiatry and Behavioral Sciences, University of California San Francisco (UCSF) School of Medicine, San Francisco, CA USA; 6 Department of Psychology, University of California Los Angeles, Los Angeles, CA USA; 7 Department of Biochemistry and Biophysics, University of California San Francisco (UCSF) School of Medicine, San Francisco, CA USA; 8 Office for Psychiatry and Habilitation, Psychiatry Research Skåne, Region Skåne, Lund, Sweden

**Keywords:** Predictive markers, Depression

## Abstract

Mitochondrial dysfunction has been implicated in major depressive disorder (MDD). A measure of mitochondrial respiratory chain (RC) enzymatic activity—the Mitochondrial Health Index (MHI)—has previously been found to correlate with stress and emotional states in caregivers. We here report mitochondrial RC activities, mitochondrial DNA copy number (mtDNAcn), and the composite MHI in unmedicated and somatically healthy subjects with MDD (*n* = 47) and healthy controls (HC) (*n* = 11). We also explore, in a subset of the MDD sample (*n* = 33), whether these markers are associated with response to 8 weeks of SSRI treatment. Mitochondrial RC complexes I, II, IV, citrate synthase (CS), mtDNAcn, and the MHI were assayed in peripheral blood mononuclear cells. Treatment response was defined as >50% decrease on the 25-item Hamilton Depression Rating Scale (HRDS-25). There were no significant differences in MHI or any of the mitochondrial markers between MDD subjects and HCs. Compared to SSRI nonresponders, SSRI responders had significantly higher baseline mitochondrial content markers CS (*p* = 0.02) and mtDNAcn (*p* = 0.02), and higher complex I activity (*p* = 0.01). Complex II activity increased significantly over treatment, irrespective of clinical response (*p* = 0.03). Complex I activity decreased in responders (*n* = 9), but increased in nonresponders (*n* = 18) (group x time interaction, *p* = 0.02). Absolute treatment-associated change in HDRS-25 scores correlated significantly with change in complex I activity between baseline and week 8 (*r* = 0.47, *p* = 0.01). Although mitochondrial markers did not distinguish MDD from controls, they did distinguish SSRI responders from nonresponders. If larger studies validate these mitochondrial differences, they may become useful biomarkers and identify new drug targets.

## Introduction

Major Depressive Disorder (MDD) is a prevalent debilitating condition, but its overall pathophysiology remains unknown. Further, the extent to which it is purely a “mental illness” or a “brain disease,” as opposed to a disorder with peripheral somatic pathologies is uncertain [1]. Apart from its psychiatric and behavioral manifestations, MDD is associated with early mortality and increased risk for several somatic conditions [2], raising the possibility of systemic pathologies, perhaps even cellular disturbances, such as telomere length shortening, inflammation, oxidative stress, and mitochondrial dysregulation [3–8].

Mitochondria generate ATP by oxidative phosphorylation, but also have a number of other vital functions including regulating apoptosis, reactive oxygen species, steroidogenesis, inflammatory and anti-inflammatory effects, and others [9–11]. The brain is highly dependent on mitochondrial functioning, as it is the largest consumer of oxygen in the body [12, 13]. Individuals with primary mitochondrial cytopathies have a very high risk of developing psychiatric disorders, particularly MDD [14, 15]. This and other evidence have suggested a link between mitochondrial function and psychiatric symptoms, including MDD and other conditions [14, 16, 17].

While mitochondrial function may indeed be related to the pathophysiology of MDD [18], the extent to which this involves core mitochondrial enzymatic dysregulation, and whether it has clinical implications for MDD, are incompletely understood. Only a few studies have investigated mitochondrial function by quantifying electron transport chain enzymatic activity in MDD subjects. One of these studies found that activity of cytochrome c oxidase (COX)—also known as Complex IV—a large transmembrane multi-protein enzyme in the electron transport chain, measured noninvasively using near-infrared spectroscopy, was lower in the prefrontal cortex in MDD compared to controls and inversely correlated with depression severity [19]. In this small-scale pilot study, some of the patients were receiving antidepressant medication, calling for replication in larger and medication-free cohorts. Another study assessed mitochondrial adenosine triphosphate (ATP) production rate (MAPR) in biopsied muscle tissue from patients with MDD (who also had at least three comorbid medical conditions common in mitochondrial diseases) and found lower MAPR in MDD subjects compared to controls [20], suggestive of impaired mitochondrial efficiency in MDD. A recent meta-analysis, including only a small number of MDD studies with generally small sample sizes, found some evidence of complex I (NADH dehydrogenase) deficiency in certain brain regions in MDD, but no evidence of changes in complex IV (COX) [21]. The inclusion of medicated patients in such studies may have introduced bias [21], again highlighting the need to investigate mitochondrial markers also in unmedicated MDD patients.

Among the different methods used for assessing mitochondrial content in leukocytes, mtDNA copy number (mtDNAcn) is among the most widely used [8, 22–27]. The number of mtDNA copies per cell in leukocytes reflects mtDNA replication and degradation and has been used to indirectly reflect mitochondrial biogenesis [28–30], but it does not reflect mitochondrial function per se [31]. Total cellular mitochondrial energy production capacity is determined by both mitochondrial content (the number of mitochondria in a cell) and the mitochondrial functional capacity (the energy production capacity of each mitochondrion). To disentangle these contributors and identify the molecular nature of a potential mitochondrial perturbation, a mitochondrial health index (MHI) was developed to assess mitochondrial functional capacity in human leukocytes [32]. This metric integrates nuclear and mitochondrial DNA-encoded respiratory chain (RC) enzymatic activities and mtDNAcn into an index that reflects mitochondrial RC capacity on a per-mitochondrion basis. The MHI was previously found to be low among high-stressed caregivers compared to controls, and the MHI was also associated with mood states in this group [32]. While these findings are novel and interesting, the MHI has not, to date, been applied to psychiatric disorders and its clinical relevance remains unknown.

In this study, we report activities of mitochondrial complexes I, II, IV, mtDNAcn, citrate synthase (CS), and the composite MHI in peripheral blood mononuclear cells (PBMCs) of unmedicated MDD subjects and healthy controls. We also explored, in a subset of the present MDD sample, whether these markers (prior to antidepressant treatment as well as changes with treatment) predicted response to SSRI antidepressant treatment.

## Methods and materials

### Ethics statement

The Investigational Review Board (IRB) of the University of California, San Francisco (UCSF), approved the study protocol (UCSF IRB # 10-00825). All study participants gave written informed consent to participate in this study and were compensated for participating. The study was pre-registered at ClinicalTrials.gov (identifier NCT00285935).

### Recruitment procedures

Unmedicated MDD outpatients (*n* = 47) and healthy controls (*n* = 11) were recruited by flyers, bulletin board notices, Craigslist postings, newspaper ads, and, in the case of MDD subjects, clinical referrals.

### Inclusion criteria

Individuals aged 21–50 years, who met DSM-V criteria for MDD, and who had a 17-item Hamilton Depression Rating Scale (HDRS) [33] score of ≥17, or a score of ≥20 on the 25-item HDRS (to account for subjects with greater atypical symptom severity) were eligible for the study. All diagnoses, including MDD, were established using the Structured Clinical Interview (SCID) and were verified in a separate diagnostic evaluation by a Board-certified psychiatrist. The Perceived Stress Scale (PSS) [34] was used to measure the experience of psychological stress in the past month and the 10-item Adverse Childhood Experiences (ACE) questionnaire was used to assess adverse childhood experiences [35].

### Exclusion criteria

MDD subjects were excluded if they met the DSM-IV criteria for any of the following: (i) bipolar disorder, (ii) alcohol or substance abuse within the preceding six months, (iii) PTSD or an eating disorder within 6 months of entering the study, and (iv) any history of psychosis outside of a major depressive episode, or the presence of any psychotic symptoms during the current major depressive episode. Potential healthy controls were excluded for any lifetime history of DSM-IV Axis-I diagnoses. All study participants (MDD and controls) were free of acute illnesses or infections, inflammatory disorders, neurological disorders, or any other medical conditions considered to be potentially confounding (e.g., cancer, HIV, diabetes, history of cardiovascular disease or stroke, etc.), as assessed by medical history, physical examinations, and blood screening, including electrolytes, lipids profile, liver, kidney, thyroid function tests, etc. All subjects were free of psychotropic medications (including antidepressants), hormone supplements, steroid-containing birth control, or other potentially interfering medications for at least 6 weeks prior to enrollment in the study. For the MDD subjects, short-acting sedative-hypnotics were allowed as needed up to a maximum of three times per week, but none within five drug half-lives prior to ratings or blood draws in the study. All subjects had to pass a urine toxicology screen for drugs of abuse (marijuana, cocaine, amphetamines, PCP, opiates, methamphetamine, tricyclic antidepressants, and barbiturates) and a urine test for pregnancy in women of child-bearing age on the same day as the blood draw and clinical ratings.

### Blood sampling

Venipuncture was performed at approximately 10:00 a.m. at the UCSF Clinical and Translational Science Institute, after 12-h of fasting (except water) and seated relaxation for at least 30 min. Plasma was collected into a lavender EDTA Vacutainer tube. Tubes were spun at 1500×*g* for 10 min at 4 °C, and plasma was removed, aliquoted into tubes, and frozen at −80 °C until assayed. PBMCs were prepared from whole blood by ficoll centrifugation [36] and were frozen at −80 ^o^C.

### SSRI treatment in the MDD subgroup

Thirty-three of the MDD subjects underwent 8 weeks of protocol-based open-label outpatient treatment with an SSRI antidepressant. In order to limit the range of potential mechanisms of action of antidepressants, the choice of medication was limited to an SSRI. The decision for the specific SSRI prescribed was based on clinical information such as medical history, family history, prior medication history, subject preference, and potential side effects. The primary outcome measure was the severity of depressive symptoms, assessed by means of the 25-item HDRS [37], which was repeated at the end of treatment (week 8). Antidepressant responders were defined as those with >50% improvement on the HDRS-25. The HDRS-25, rather than the 17-item HDRS, was used to define response since the former scale includes several items (e.g., weight gain, loss of energy/fatigue) that might be linked to mitochondrial function [38, 39].

Twelve subjects were treated with sertraline (mean ± SD dose in mg = 131 ± 55), six with fluoxetine (dose in mg = 30 ± 11), four with citalopram (mean + SD dose in mg = 34 + 13), and 11 with escitalopram (mean + SD dose in mg = 18 + 5). Medication dosages were increased over the course of treatment per prespecified protocol as tolerated and as warranted by clinical response. MDD subjects had blood drawn at weeks 4 and 8 for serum antidepressant concentrations to assess medication compliance. In each of these subjects, plasma antidepressant concentrations were in the reported clinical range for that antidepressant, suggesting excellent compliance.

### Rationale for calculating the MHI

The MHI was computed by integrating three enzymatic measures of respiratory chain capacity and two mitochondrial content features, as described previously [40]. Briefly, PBMC pellets were mechanically homogenized with tungsten beads in a homogenization buffer to release individual enzymes. The homogenate was then used to quantify citrate synthase (CS), NADH dehydrogenase (complex I), succinate dehydrogenase (SDH, complex II), and cytochrome c oxidase (COX, complex IV) using kinetic spectrophotometric assays. The specific activity for each enzyme was obtained by calculating the slope (first derivative) of the optical density change, and subtracting nonspecific activity detected in the presence of specific inhibitors for each complex, or in the absence of the rate-limiting reaction substrate. All assays were performed in triplicates, at 30 ^o^C, and final values were normalized on a per-cell basis using the qPCR-based estimates of cell numbers for each biological sample as described in ref. [32].

Mitochondrial and nuclear genome abundances were determined from 20 ul of the same homogenate used for enzymatic measurements lysed to extract genomic material (nDNA and mtDNA). The resulting lysate was used as the DNA template for two different Taqman multiplex assays for ND1 (mtDNA) and B2M (nDNA), and for COX1 (mtDNA) and RnaseP (nDNA), as previously described in details [32]. mtDNAcn was estimated using the ΔCt method, subtracting the mtDNA Ct from nDNA Ct for each amplicon pair (ND1:B2M and COX1:RNaseP), and then multiplying by 2 to account for the diploid nuclear genome. The mtDNAcn values from both assays were averaged to obtain the final mtDNAcn value for each participant.

To compute the MHI, the five mitochondrial features were mean-centered so that each parameter contributes an equal weight to the equation. Combining three features (complexes I, II, and IV) as a numerator, divided by two content features (CS and mtDNAcn) as a denominator produces a quantitative index of mitochondrial energy production capacity, or “quality” on a per cell mitochondrion basis, where a value of 100 represents the average of the cohort, and values >100 and <100, respectively, indicate higher and lower respiratory chain capacity on a per-mitochondrion basis. Previously, the composite MHI exhibited a higher predictive ability of caregiver (chronic stress) status than any of the individual MHI components alone [32].

### Statistics

The Statistical Package for the Social Sciences (SPSS) v.22 (IBM Corp., Armonk, NY) was used for statistical calculations. All tests were two-tailed with an alpha = 0.05. Non-normally distributed variables were log10 transformed. Group differences were tested by independent sample *t*-tests or Chi-square. Correlations were tested using Pearson’s *r*. Paired samples t-tests were used to test change in biomarkers pre- to posttreatment with SSRI. We also performed repeated-measures ANOVAs (within and between-subjects design) to test time (biomarker) × group (responder/nonresponder status) interactions on the changes in biomarkers between baseline and week 8 in SSRI responders and nonresponders. Binary logistic regression (with MDD/control and SSRI-responder/SSRI-nonresponder as dependent variable) were performed for multivariate analyses, adjusting for age and gender. Twelve MDD patients and three controls had missing baseline data on one or more mitochondrial enzymes due to technical reasons. For the purpose of calculating the MHI for these individuals, missing data were imputed using the group average. Sensitivity analyses were carried out for all calculations involving the MHI, including (i) only those subjects with full data and (ii) all subjects (including those for whom imputed data was used to replace missing values). All samples were collected over a 7-year period, and we observed a substantial effect of storage time on all enzyme activities, ranging from 5–12% activity loss per year. Therefore, all mitochondrial markers were adjusted for storage time by removing the variance (regression slope) attributable to time since collection. This is the first clinical study to test the association between these mitochondrial markers and antidepressant treatment response, thus these analyses should be considered exploratory and were not adjusted for multiple comparisons [41].

## Results

### Demographic characteristics

Demographic characteristics of MDD subjects and controls are presented in Table 1. There were no significant group differences between MDDs and HCs with regard to age, sex, smoking, or BMI. There were no significant differences in age, BMI, or smoking between responders and nonresponders, but responders were more likely to be females.

**Table 1:** Demographic characteristics of subjects with MDD and controls.

	MDD *N* = 47	Controls *N* = 11	*P* value	Responders (*n* = 11)	Nonresponders (*n* = 22)	*p* value
Sex (f/m)	26/21	6/5	0.96	9/2	9/13	0.03
Age (Years; mean + SD)	35.0 ± 10.7	35.1 ± 11.1	0.99	39.0 ± 9.4	36.4 ± 11.8	0.52
BMI (kg/m^2^; mean + SD)	25.1 ± 4.0	23.9 ± 2.9	0.33	25.4 ± 5.1	24.8 ± 4.0	0.72
Cigarettes smoked per day (mean ± SD)	0.19 ± 0.74	0 ± 0	0.42	0.28 ± 0.90	0.14 ± 0.64	0.61

Age was significantly correlated with mitochondrial RC complex IV (*r* = −0.29, *p* = 0.04). MHI was positively correlated with BMI (*r* = 0.28, *p* = 0.03). None of the mitochondrial biomarkers were significantly associated with sex or smoking (data not shown). MHI was not significantly correlated with ACE, PSS score, or the HDRS-25 suicidality item (all *p* > 0.2).

### Mitochondrial markers in MDD subjects and controls

There were no significant differences in MHI or any of the individual mitochondrial markers between MDD subjects and healthy controls (Table 2). These results did not change when only including those with full MHI data or after adjusting for age and gender. There were no significant correlations between any of the biomarkers and depression severity ratings based on the 25-item HDRS (all *p* > 0.05).

**Table 2:** Mitochondrial markers in MDD and controls.

	Unmedicated MDD *N* = 47	Controls *N* = 11	*P*- value	Effect size (Cohen’s *d*)	Responders, Baseline (*n* = 11)	Nonresponders, Baseline (*n* = 22)	*p* value	Effect size (Cohen’s *d*)
MHI (mean ± SD)	99.4 ± 14.4	95.6 ± 10.5	0.41	0.30	99.2 ± 10.9	95.4 ± 13.7	0.42	0.31
CS (mean ± SD)	45.5 ± 13.8	51.0 ± 31.4	0.59	0.23	50.1 ± 8.3	38.2 ± 13.7	0.02	1.05
Complex I (mean ± SD)	5.8 ± 1.9	5.8 ± 2.9	0.84	0.00	6.8 ± 1.8	5.0 ± 1.8	0.01	1.00
Complex II (mean ± SD)	76.0 ± 21.0	79.6 ± 33.6	0.85	0.13	79.0 ± 23.0	69.3 ± 19.2	0.27	0.46
Complex IV (mean ± SD)	10.9 ± 3.7	10.8 ± 5.6	0.79	0.02	10.1 ± 3.6	9.9 ± 2.0	0.76	0.07
mtDNAcn (mean ± SD)	437.3 ± 62.7	435.1 ± 106.7	0.93	0.03	464.6 ± 55.0	416.1 ± 54.4	0.02	0.89

Raw values are presented in the table, although *t*-tests using log-transformed data were used. Missing values for CS, *n* = 4 (four MDD, all nonresponders); complex I, *n* = 3 (three MDD, one responder, one nonresponder, one non-treated); complex II, *n* = 8 (five MDD, three nonresponders, one responder, one non-treated; three controls); complex IV, *n* = 6 (six MDD, three nonresponders, two responders, one non-treated). The group differences in MHI remained nonsignificant in sensitivity analyses including only those with full data (*p* > 0.53). Enzymatic activities are in mmol/min/106 cells, and mtDNAcn are copies per cell.

*MHI* mitochondrial health index, *CS* citrate synthase, *mtDNAcn* mitochondrial DNA copy number.

### Mitochondrial markers in SSRI responders and nonresponders

Pretreatment, SSRI responders had significantly higher mtDNAcn (*p* = 0.02), higher CS activity (*p* = 0.02), and higher RC complex I activity (*p* = 0.01) compared to SSRI nonresponders. These findings were also significant after adjusting for age and gender (all *p* < 0.05).

In all treated MDD subjects, Complex II increased significantly between baseline and after 8 weeks of SSRI treatment (*p* = 0.03). None of the other mitochondrial markers or MHI changed significantly with treatment in all treated subjects (Table 3). The results did not change for MHI when only those subjects with full data were included.

**Table 3:** Pre- and posttreatment mitochondrial markers in all MDD subjects undergoing 8 weeks of SSRI treatment.

	Pretreatment, baseline	Posttreatment, after 8 weeks of SSRI treatment	*P* value	Effect size (Cohen’s *d*)
MHI (mean ± SD)	70.3 ± 12.9	65.4 ± 15.3	0.56	0.10
CS (mean ± SD)	42.3 ± 13.2	48.2 ± 15.3	0.07	0.35
Complex I (mean ± SD)	5.8 ± 2.0	5.9 ± 2.5	0.77	0.05
Complex II (mean ± SD)	73.2 ± 20.8	86.6 ± 28.9	0.03	0.46
Complex IV (mean ± SD)	9.8 ± 2.7	11.5 ± 5.1	0.20	0.29
mtDNAcn (mean ± SD)	433.5 ± 59.1	460.5 ± 94.6	0.13	0.26

Raw values are presented in the table, although paired *t*-tests using log-transformed data were used (except for MHI which was normally distributed). Enzymatic activities are in mmol/min/106 cells, and mtDNAcn are copies per cell. Full data from both time points (baseline and week 8) were available for 32 subjects (MHI, including imputed values), 28 subjects (CS), 27 subjects (Complex I), 25 subjects (Complex II), 22 subjects (Complex IV), and 32 subjects (mtDNAcn).

**Fig. 1:**
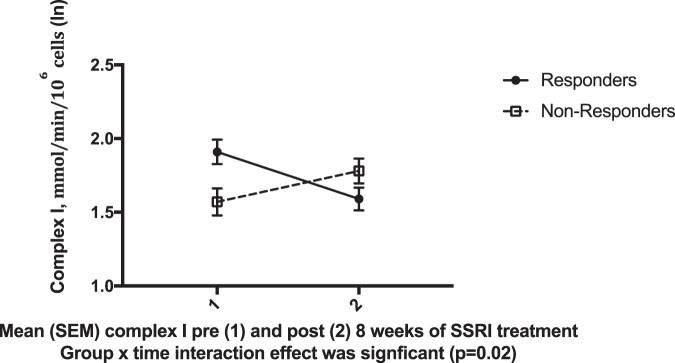
Change in PBMCs RC Complex I activity between baseline and 8 weeks of SSRI treatment SSRI responders and nonresponders. Complex 1 activity is plotted on the y-axis and time point (baseline and week 8) on the x-axis.

**Fig. 2:**
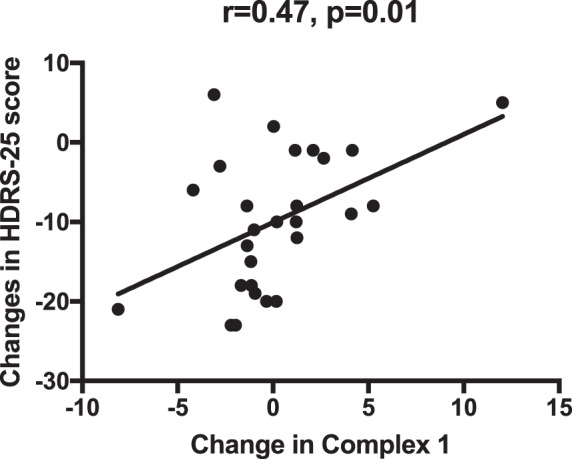
Change in HDRS-25 scores correlated significantly with change in Complex I activity between baseline and 8 weeks of SSRI treatment. A negative score indicates a greater decrease over time, delta scores were computed by subtracting the week 8 value from the baseline value for HDRS-25 and Complex I respectively. This correlation was also significant using nonparametric statistics (rho = 0.4, *p* = 0.04).

Complex I decreased in SSRI responders (*n* = 9), but increased in nonresponders (*n* = 18) (group × time interaction, *p* = 0.02, Fig. 1). There were no other significant time × group effects for any of the other biomarkers including MHI (all *p* > 0.05). Absolute change in HDRS-25 correlated significantly with an absolute change in Complex I activity between baseline and week 8 (*r* = 0.47, *p* = 0.01) (Fig. 2), such that a decrease in Complex I activity over treatment was associated with greater improvement on the HDRS-25.

## Discussion

We assessed the activity of RC enzymes, mtDNA, and the composite MHI in blood samples from unmedicated depressed patients and healthy controls. Although there were no significant between-group differences in any markers of mitochondrial content or RC activity, we did note significant associations between mitochondrial features and antidepressant response. Specifically, we found evidence that higher baseline (pre-SSRI treatment) mtDNAcn, as well as greater activity of CS and complex I, were associated with a better antidepressant response to an SSRI. Moreover, a greater treatment-associated decrease of Complex I was also associated with better SSRI response. Finally, complex II activity increased with SSRI treatment, irrespective of clinical response.

We found associations between certain mitochondrial features and antidepressant treatment response, whereas other features were not associated. Dysfunctional mitochondrial enzymes may promote oxidative stress in various disorders [42], and increased levels of oxidative stress, in turn, have been associated with poorer SSRI treatment response [7]. Additional mechanisms for decreased mitochondrial function hindering antidepressant efficacy have also been proposed, including inflammation, apoptosis decreased activation of downstream neurotransmitter signaling, decreased vesicle release, and, possibly most importantly, decreased neuronal plasticity [43]. We found that higher baseline complex I enzyme activity, and a greater decrease over treatment, was associated with superior antidepressant treatment response. Mitochondrial RC complex I is the first enzyme of the mitochondrial RC and coordinates the transfer of electrons from Krebs cycle-derived NADH to coenzyme Q10, thus generating the proton gradient across the mitochondrial inner membrane, resulting in ATP production [44]. It is also a major site of reactive oxygen species (ROS) production within the mitochondria, which is modulated by a number of factors including membrane potential production [45]. Therefore, activity is not directly related to ROS production. RC complex I is critical for cellular functions, and deficiencies or dysfunction in complex I activity has been implicated in neurodegenerative disorders such as Alzheimer’s and Parkinson’s disorder [46]. Post-mortem studies have also reported lower complex I levels in brain tissue from MDD patients compared to controls [21]. RC complex 1 may suppress inflammation [47], highlighting another important mechanism linking mitochondrial function to an antidepressant response, given that systemic low-grade inflammation has been associated with lower SSRI efficacy [48–50]. Future mechanistic studies are needed to better understand how inflammation and mitochondrial dysfunction may interact to contribute to depression treatment resistance. Although this is the first clinical study, to the best of our knowledge, to investigate the association between antidepressant treatment response and specific mitochondrial enzymes, several preclinical studies have demonstrated an effect of antidepressants on mitochondrial function [43]. In a series of rodent experiments, Agostinho et al. [51, 52] reported several brain region-specific effects on mitochondrial enzyme activity of chronic and acute fluoxetine administration. They found that complex I activity decreased in the prefrontal cortex, but not in the hippocampus or the striatum, after chronic fluoxetine treatment. Complex IV activity decreased in the hippocampus, but not in the other brain regions, following chronic fluoxetine administration, and no significant effects were seen for complex II or CS [51]. In our sample, complex II increased with SSRI treatment, irrespective of clinical response. Partly in line with Agostinho et al., complex I activity decreased with SSRI treatment, but only in those patients who responded to treatment; in treatment nonresponders, on the other hand, complex I activity increased with SSRI treatment. These findings suggest that the relationship between antidepressant effect and mitochondrial function is complex, and more studies with larger samples are needed to elucidate this. Moreover, the basis underlying the specificity of our findings to complex I relative to complex IV (which similar to complex I is partially mtDNA encoded) and complex II (nuclear-encoded), is unclear and will require independent validation.

Our primary hypothesis was that unmedicated MDD subjects would have a lower MHI than controls, reflecting lower mitochondrial bioenergetic capacity. The rationale behind this was based on (i) the finding that individuals suffering from mitochondrial cytopathies, which decreases RC capacity on a per-mitochondrion basis, or MHI, often display depressive symptoms [14], (ii) several animal and human studies linking decreased mitochondrial function to depressive symptoms [14, 18, 20, 43, 53], and (iii) greater self-reported positive mood in women predicted higher MHI the following days [32]. We did not find a significant difference in MHI, or in any of the individual mitochondrial enzymes, between MDD subjects and controls. We speculate that it is possible that MDD is associated with various compensatory mechanisms that counteract the hypothesized decrease in mitochondrial respiratory function. Also, it is possible that the cross-sectional design of the study may not have been appropriate to capture potential dynamics of mitochondrial function over time, where mitochondria may respond to mood states within hours to days [32] and may change from week to week [40]. It is also possible that mitochondrial function is not associated with the heterogeneous disorders of MDD per se, but rather certain aspects of MDD, including treatment response to antidepressants. Finally, our sample size may have been too small to detect statistically significant differences between groups, although the effect sizes of the differences between the groups somewhat argues against this.

A strength of the present study is that we have used a multivariate approach to profile potential mitochondrial alterations or recalibrations, including both mitochondrial content (CS and mtDNAcn) and RC capacity, in leukocytes. Advantages of using a composite score such as the MHI include its ability to provide an integrated representation of mitochondrial function from an easily accessible blood sample, including samples that have been previously frozen. While this is an important strength of our paper, we also note that the effects of stress and allostatic load on mitochondrial function and structure (i.e., mitochondrial allostatic load) may manifest in multiple aspects of mitochondrial behavior including DNA integrity, gene expression, protein composition, respiration, and signaling functions [54]. Future studies would gain by assessing a more comprehensive panel of mitochondrial behaviors to further our understanding of the relationship between mitochondrial health and depression. Additional strengths of our study are that: (i) depressed subjects were carefully evaluated and were unmedicated for at least six weeks prior to blood draws and were free from somatic illnesses and substance abuse; (ii) control subjects were rigorously screened for the absence of somatic and psychiatric pathologies; (iii) clinical response and adherence to the SSRI treatment protocol were closely monitored and verified. The study also has limitations, notably (i) the relatively small number of subjects, especially healthy controls and MDDs receiving antidepressant treatment (in particular, those who were “responders” to treatment), (ii) the open-label nature of SSRI treatment, although this best approximates real-world treatment conditions, and (iii) the use of PBMC cell mixtures, rather than specific purified cell types, which could have reduced our ability to detect mitochondrial alterations with greater sensitivity and specificity among particular cell populations [40]. Another limitation is that we did not have specific a priori hypotheses regarding mitochondrial component associations with SSRI treatment response, so these findings should be considered preliminary and in need of replication. While most animal studies testing the relationship between SSRI treatment and brain mitochondrial function used fluoxetine [51, 52], depressed subjects in our study were treated with a variety of SSRI agents. Although it is possible that the effect of SSRI treatment on mitochondrial function depends on the type of SSRI, we were not able to test this hypothesis given the relatively small sample size. Another important consideration when interpreting our findings is that mitochondrial health may be dynamically influenced by several factors that were not accounted for in the present study. Some of these include acute mood states [32] as well as exercise [55] and dietary habits [56]. Finally, we did not adjust the *p* value threshold for multiple comparisons which could be considered an additional limitation of the study. This is the first clinical study to test the relationship between these mitochondrial markers and antidepressant response. Thus these analyses should be considered exploratory and future, confirmatory, studies are needed to validate these findings.

In summary, our results suggest that, while the overall diminished mitochondrial respiratory capacity of PBMCs may not characterize MDD as a group, it may occur in relation to antidepressant treatment (with relatively higher complex I activity at baseline and relatively larger decreases with treatment associated with better antidepressant response). Future preclinical and clinical studies, especially longitudinal ones, will be required to ascertain the causes, consequences, and specific pathways involved in triggering mitochondrial recalibrations. A better understanding of the dynamic and complex mechanisms by which mitochondrial health is maintained, and how this may be altered in certain conditions may yield new strategies for treating groups of depressed patients or else current treatments might be used in a more targeted way. Lastly, further elucidation of these effects may clarify the nature and causes of certain comorbid systemic illnesses occurring with increased frequency in MDD and may broaden the perspective of depression beyond strictly monoaminergic theories [57].

## Supplementary information

checklist
